# Supplementary material for: Are carbon emissions trading and green financial instruments synergistic? -Comprehensive quantitative research based on content analysis
Source: PLoS One. 2024 Mar 7;19(3):e0298601. doi: 10.1371/journal.pone.0298601 (PMC10919601; doi:10.1371/journal.pone.0298601)
Supplement: S1 Data — (ZIP) [file pone.0298601.s001.zip › Data/地级市Z_F工作报告.zip!/╣π╬≈/║╙│╪╩╨/└┤╫╘╣1⁄2╓┌║┼í╛┬φ┐╦╩2╛▌═°í┐_2016.docx]

各位代表：

现在，我代表市人民政府向大会作政府工作报告，请各位代表连同《河池市国民经济和社会发展第十三个五年规划纲要（草案）》一并审议，并请各位政协委员和列席会议的同志提出意见。

一、2015年工作回顾

过去的一年，面对经济下行压力持续加大的不利影响，我们在自治区党委、政府和市委的坚强领导下，在市人大、市政协的监督和支持下，全面贯彻党的十八大和十八届三中、四中、五中全会精神，深入贯彻习近平总书记系列重要讲话精神，落实中央、自治区和市委各项决策部署，坚持稳进突围、提质发展“一个基调”，把牢“三严三实”专题教育和开展产业增量提质、民生保障提升、开发扶贫攻坚、城乡新貌新风、干部执行力提升“五大工程”提升年活动“两个抓手”，推动工业园区建设、开发扶贫创新、巴马长寿养生国际旅游区建设“三个突围”，营造抓落实重实干、抓建设促发展、抓责任敢担当、抓纪律树新风“四个风气”，推动经济、政治、文化、社会和生态文明建设“五个发展”，全市各项工作在艰难环境下取得新的进步。

（一）经济稳中提质整体向好。面对经济下行压力，我们把稳增长摆在重中之重，及时出台“48条”举措，多管齐下，精准发力，推动全市经济持续健康发展。初步统计，全市地区生产总值618.03亿元，增长4.5%；规模以上工业总产值376.43亿元，与上年持平；规模以上工业增加值136.35亿元，增长3.8%；财政收入56.13亿元，增长2.68%；固定资产投资395.69亿元，增长15.3%；社会消费品零售总额243.38亿元，增长8.8%；城镇居民人均可支配收入22751元，增长6.5%；农村居民人均纯收入6104元，增长7.7%。

（二）产业结构调整有新进展。三产比重上升2个百分点，三次产业结构从2014年的22.8:34.1:43.1调整为22.7:32.2:45.1。

工业经济平稳增长。水力发电321亿千瓦时，电力行业完成产值116亿元，增长16.3%，发电量和产值创历史新高。优质饮用水产业整合成效明显，完成产值10亿元，增长43.8%。淘汰所有立窑水泥生产线，完成年产新型干法水泥1200万吨项目布局。工业与旅游融合发展取得突破，实现工业旅游收入1000万元。丹泉酒业公司建成世界最大的天然藏酒洞，成为全国工业旅游示范点。工业园区不断壮大，完成总产值231亿元、增加值57亿元，其中，大任产业园基础设施建设加快推进，完成投资4.17亿元，入园二级公路基本贯通，鑫锋、金兴2家企业主体工程开工建设，成源、生富、华远、恒吉4家企业完成场地平整。新增规模以上企业15家，亿元企业达55家。

农业经济持续增长。粮食产量实现六连增，总产量达105万吨。特早熟、特晚熟、特优质“三特”农业规模扩大，效益提高。新增桑园2.1万亩，总面积达80万亩，鲜茧产量11.1万吨，桑园面积和鲜茧产量继续稳居全区第一，蚕桑资源综合利用经验在全国推广。牛和山羊饲养量及出栏量、内陆大水面开发养殖面积排全区首位。富硒农产品基地建设走在全区前列。凤山创建国家生态农业公园。都安、南丹、天峨各建成1个自治区现代特色农业（核心）示范区。林下经济产值42亿元，增长18.1%。建成高标准基本农田4.19万亩。农作物耕种收综合机械化率达41%。新增和恢复灌溉面积9.14万亩，改善灌溉面积17万亩。

现代服务业加快发展。实施巴马长寿养生国际旅游区基础设施建设大会战164个项目，完成投资15.5亿元。成功举办“巴马论坛——中国•东盟中医药健康旅游国际论坛”。宜州、大化、天峨先后获评“中国长寿之乡”。加大旅游品牌建设，新增国家4A级景区3家、3A级景区5家，国家四星级休闲农业示范区1家、广西四星级乡村旅游区5家。全市96项乡村旅游品牌进入国家乡村旅游“百千万”品牌榜，总数居全区前列。全市接待国内外游客1800万人次，增长16.9%；旅游总收入180亿元，增长22.8%。巴马成为“全国电子商务进农村综合示范县”试点。房地产市场稳健发展，完成投资37.9亿元，增长39.7%。金融平稳增长，2015年末，全市各项存款余额886.92亿元，全年增加72.53亿元，增长8.1%；各项贷款余额510.55亿元，全年增加39.03亿元，增长8.3%。

（三）脱贫攻坚走出新路子。全市16万贫困人口实现脱贫。持续开展“整乡推进”示范点建设，首批11个试点乡镇受益群众25万人，其中贫困人口10.3万人。十大扶贫主导产业发展取得明显成效，其中，新种核桃43万亩，总面积达216万亩，成为华南地区最大的核桃基地，入选国家首批森林生态产品生产基地。扶贫生态移民搬迁和旅游扶贫工作走在全区前列。投入6.98亿元实施《岩滩水电站水库移民遗留问题处理规划》项目1880个，发放移民口粮补助7655万元。实行“5321”扶贫帮扶机制，2.2万名干部与10多万户群众结成对子。730家企业参加“千企助百村”行动，捐款捐物1.29亿元。选派2453名工作队员开展新一轮精准识别，全面完成57万多户的入户调查工作。

（四）基础设施明显改善。交通项目建设完成投资46.24亿元，增长20%。加快建设河池至百色高速公路，累计完成投资9.25亿元；武鸣至都安、大化至巴马、环江（华山）至金城江3条二级公路改扩建工程基本完工；新建通建制村沥青（水泥）路71条666公里，建制村道路硬化率达97%；新建和硬化屯级路895条1056公里；竣工都安港区红渡作业区技改项目，开通运营红水河都安至珠三角集装箱航线；贵阳经河池至南宁客运专线项目前期工作取得实质性进展。实施左右江革命老区重大工程建设三年行动计划，建设重大项目41个总投资54.55亿元。投入3.96亿元升级改造农村电网。建成中小河流堤防42公里、防渗渠道958公里。实施旱改水项目6224亩，连续17年实现耕地占补平衡。治理地质灾害隐患点129处，保障1.2万名群众生命财产安全。

（五）城乡面貌持续改观。建设城乡新貌新风项目288项，完成投资47亿元。市中心城区基础设施建设有新改善，完成3条主干道、26条巷道改扩建；开工建设老年大学，铜厂片区、南桥片区棚户区改造等项目；基本完成城区污水收集系统管网工程建设。大力开展市中心城区“两违”和粉尘专项整治，超额完成淘汰黄标车年度任务，空气质量持续稳定达到国家二级标准。建设6个自治区百镇示范工程和5个特色名村示范点，建成10个乡镇污水处理厂。深入推进“美丽河池” 乡村建设，农村人居环境得到改善。全市森林覆盖率达68.68%。

（六）改革开放不断深化。推进行政审批制度改革，市本级行政审批项目由515项精简到159项。全面推行“三证合一、一照一码”工商登记新模式，新增企业1714户，同比增长37.9%；新增注册资本29.53亿元，同比增长46.8%。推动国资国企整合重组，增强国有资本投融资能力，累计融资22.87亿元。市、县两级成立不动产登记机构。开展县级公立医院、供销合作社、乡镇机构“四所合一”等改革试点。区域合作步伐加快，启动河池（天峨）•黔南州（罗甸）桂黔跨省经济合作产业园项目前期工作，建立滇黔桂红水河库区安全协调工作机制。招商引资成效明显，内资到位269亿元，增长8.1%；全口径实际利用外资1.2亿美元，增长20%。第十二届中国—东盟博览会签约项目45个，总投资250亿元。

（七）社会事业全面发展。全市财政民生支出218.43亿元，占财政总支出84.2%。教育、文化、体育、卫生计生等各项社会事业取得新成效。学前三年毛入园率达70.8%、义务教育巩固率达88.8%、高中阶段毛入学率达80%。出品孝道文化动画片《攀霞降魔记》，获得西安第四届国际原创动漫大赛特别奖提名。成功举办都安60周年县庆、第十六届铜鼓山歌艺术节和市第五届运动会等重大活动。全民健身活动蓬勃开展，参加自治区第十三届运动会获得佳绩。人口计生工作不断加强，人口自然增长率7.09‰。基本建成覆盖城乡的社会保障体系，医疗保险实现全区“一卡通”，新型农村合作医疗参合率达99.53%；基本养老保险金发放率100%；五项社会保险参保人数112.83万人，基金征缴收入16.14亿元。城镇新增就业2.35万人，农村劳动力转移就业新增6.57万人次。新开工保障性住房1.76万套，分配入住9767户。农村危房改造5.8万户。解决33.7万农村群众饮水不安全问题。应急管理体制机制不断健全。深入落实“党政同责、一岗双责”责任体系，安全生产形势持续向好。食品药品监管全面加强。创新社会管理工作取得新突破，“天峨经验”在全国推广，群众安全感持续保持全区前列。

（八）双拥共建力度加大。积极创建全国双拥模范城，在全区率先出台《进一步加强拥军优属工作，提升军人荣誉感的规定》，得到广西军区肯定。不断加强国防教育、国防后备力量建设。驻军部队大力支持地方发展，出色完成大化六也乡抗洪救灾等急难险重任务。市人民防空办公室获得“广州军区人防机关准军事化建设先进单位”荣誉称号。

（九）自身建设不断加强。严格落实中央八项规定和国务院“约法三章”。扎实开展“三严三实”专题教育，切实提高政府法治建设。认真执行市人大及其常委会的决定决议，自觉接受人大、政协和社会监督，办理人大代表建议和政协提案176件，办复率100%。完善依法行政制度体系，建立政府法律顾问、重大行政决策专家咨询论证和风险评估制度。依法行政考核排名居全区前列。公布首批20个部门权力清单和责任清单。在全区率先建成市县乡村四级联动政务服务体系。加大行政监察和审计监督力度，推进党风廉政建设和反腐败工作，群众对党风廉政建设和反腐败工作满意度在全区排位由第5位上升至第3位。

统计、司法行政、税务、公共资源交易、宗教、外事侨务、机关事务、消防、保密、检验检疫、档案、地方志、通信、海事、气象、地震、水文等工作迈出新步伐，妇女儿童、老干部、老龄、残疾人保障等事业取得新进展，工会、共青团、妇联等群团组织在经济社会发展中发挥了重要作用。驻河池中直、区直各单位取得新成绩，多个单位、部门荣获全国或自治区级荣誉。

2015年是“十二五”的收官之年。“十二五”期间，我们走过了极不平凡的历程，战胜了各种艰难险阻，全市呈现经济稳健运行、社会和谐稳定、人民安居乐业的良好局面。减贫人口、森林覆盖率、九年义务教育巩固率等约束性指标全面完成目标任务；地区生产总值、财政收入、固定资产投资等预期性指标保持平稳增长。

各位代表！成就令人振奋，经验弥足珍贵。我们更加深刻地认识到，要全面建成小康社会，必须坚持打基础与利长远并重，牢固树立“只要惠及子孙，功成不必在我”的理念，定准方向，把准规律，选准路径，一茬接着一茬干，推动河池科学发展；必须坚持转型升级与增量提质并重，以质量和效益为中心，深入开展以环境倒逼机制推动产业转型升级攻坚战，实现产业提质与增量协同发展；必须坚持精准脱贫与改善民生并重，全力以赴打好精准脱贫攻坚战，切实保障和改善民生，着力解决好群众最关心最直接最现实的利益问题；必须坚持科学开发与加强保护并重，保护好、利用好、开发好生态资源，把生态优势转化为发展优势，走绿色发展之路；必须坚持依法行政与优质服务并重，进一步简政放权，转变职能，不断提高服务质量和行政效率，营造良好的发展环境。

各位代表，回顾过去的工作，我们深深感到成绩来之不易。这是市委正确领导、科学决策的结果，是历届班子打下良好基础的结果，是人大、政协及社会各界积极帮助、大力支持的结果，是全市人民群策群力、共克时艰的结果。在此，我谨代表市人民政府，向全市各族人民，向人大代表、政协委员，向各民主党派、工商联和社会各界人士，向驻河池中直、区直各单位和驻河池部队，向所有关心支持河池发展的朋友们，致以崇高的敬意和衷心的感谢！

在看到成绩的同时，我们也清醒地认识到，由于历史及自然地理条件等原因，河池还面临着一些困难和问题：一是发展底子薄，经济总量小，在经济下行压力加大的形势下，发展新动力不足；二是交通等基础设施条件较差，发展环境约束明显；三是全市仍有68万贫困人口，精准脱贫攻坚任务艰巨；四是投资环境需进一步优化，个别部门和个别干部服务意识不强、效率不高，抓落实不到位的现象依然存在。对当前面临的这些问题和困难，我们将以对人民高度负责的态度，求真务实、勇于担当，积极采取各种有效措施，努力加以克服和解决，不辜负全市各族人民的期望。

二、2016年工作重点

今年是“十三五”的起步之年，也是全面建成小康社会决胜阶段的开局之年。根据《中共河池市委员会关于制定国民经济和社会发展第十三个五年规划的建议》，市人民政府组织编制了《河池市国民经济和社会发展第十三个五年规划纲要（草案）》，进一步细化了发展目标，科学谋划了发展布局。

《纲要》提出“十三五”时期主要经济社会发展目标是：经济持续健康发展，地区生产总值年均增长7%；全面实现精准脱贫，坚决打赢脱贫攻坚战，到2020年，确保全市现行标准下68万农村贫困人口实现脱贫，确保10个贫困县（区）（含“天窗县”）、684个贫困村脱贫摘帽；人民生活水平显著提高，城乡居民收入差距进一步缩小。

做好今年工作，对实施“十三五”规划开好局起好步，意义重大、影响深远。当前，国家正大力实施脱贫攻坚战、左右江革命老区振兴规划、滇桂黔石漠化片区区域发展与扶贫攻坚规划、珠江—西江经济带发展规划以及“一带一路”等战略部署，加大对革命老区、贫困地区、少数民族地区的扶持力度，为我市加快经济社会发展带来了前所未有的机遇。我们要树立强烈的机遇意识和追赶意识，切实学深学透这些政策，用活用好这些政策，凝心聚力，克难攻坚，推动经济社会发展再上新台阶。

今年政府工作总体要求是：高举中国特色社会主义伟大旗帜，全面贯彻党的十八大和十八届三中、四中、五中全会精神，以马克思列宁主义、毛泽东思想、邓小平理论、“三个代表”重要思想、科学发展观为指导，深入学习贯彻习近平总书记系列重要讲话精神，按照“五位一体”总体布局和“四个全面”战略布局，坚持发展第一要务，以提高经济发展质量和效益为中心，牢固树立和贯彻落实创新、协调、绿色、开放、共享的发展理念，坚持稳进突围、提质发展“一个基调”，用好深化“三严三实”学习教育和开展“五大工程巩固年”活动“两个抓手”，推动精准脱贫攻坚、园区建设攻坚、巴马长寿养生国际旅游区建设攻坚“三个攻坚”，营造抓改革促发展、抓落实重实干、抓责任敢担当、抓法纪树新风“四个风气”，促进经济、政治、文化、社会、生态文明“五个发展”，加快建设幸福新河池，与全国全区同步全面建成小康社会。

今年经济社会发展主要预期目标是：全市地区生产总值增长7%，规模以上工业增加值增长8%，财政收入增长6.5%，固定资产投资增长18%，社会消费品零售总额增长9%，外贸进出口总额增长5%，全体居民人均可支配收入增长8%，其中农村居民人均可支配收入增长8.5%，居民消费价格指数控制在3.5%左右，城镇登记失业率控制在4.5%以内，人口自然增长率控制在9‰以内，节能减排指标控制在自治区下达的目标范围内。

为实现今年工作目标任务，要重点做好以下十项工作：

（一）奋力抓好脱贫攻坚，加快精准脱贫步伐。

河池是广西乃至全国精准脱贫攻坚主战场。我们一定要把扶贫开发作为最大的政治责任、最大的民生工程、最大的发展机遇，横下一条心，坚定愚公移山志，采取超常规举措，打好精准脱贫攻坚战。今年，全市减少农村贫困人口20万人；684个“整村推进”贫困村农民人均纯收入达3800元；全面完成精准识别建档立卡，建立扶贫大数据动态管理平台。

精准实施“七个一批”脱贫行动。一是产业发展脱贫。继续抓好核桃、糖料蔗、桑蚕、蔬菜、食用菌、经济林、特色水果、草食动物生态养殖、水产养殖、特色畜禽养殖等十大扶贫主导产业，实现产业扶持贫困户全覆盖。其中，新种核桃20万亩，认真抓好管护工作。加大旅游扶贫力度，实施乡村旅游富民工程，重点扶持24个贫困村发展旅游业，创建50个以上旅游扶贫示范点，带动1.7万贫困人口脱贫。二是转移就业脱贫。帮扶引导外出务工60万人以上。三是移民搬迁脱贫。巩固移民搬迁有土安置和无土安置工作成果，对“一方水土养不活一方人”的贫困地区实施易地搬迁发展。重点推进大化、都安、环江、罗城、天峨等移民安置项目建设。全市搬迁安置移民5万人。四是生态补偿脱贫。管护好1952万亩生态公益林，兑现2.8亿元补偿资金。五是教育扶智脱贫。全面落实各项教育精准扶贫扶持政策。实施“9＋3”免费教育计划，对完成九年义务教育未能继续升学的贫困家庭子女全部纳入三年免费中等职业教育范畴。落实中职学生免学费政策，资助贫困学生1.4万人。六是医疗救助脱贫。对因病致贫、因病返贫的农村贫困人口，纳入重特大疾病救助范围。实施大病保险，对新农合补偿后个人自付超过6000元的参合农民予以保险理赔。七是社保兜底脱贫。推进农村低保与扶贫开发有效衔接，将符合低保条件的贫困户全部纳入最低生活保障范围。提高城乡低保补助标准，全市城乡低保对象月人均补差水平分别达到280元和125元以上。

加强贫困村屯基础设施建设。深入推进贫困村屯路、水、电、网络等基础设施建设，新建屯级路180条300公里以上，硬化屯级路300条360公里以上；新建、改扩建713个村屯人畜饮水工程，巩固提升18万农村人口饮水安全；解决41个自然村未通电问题；行政村互联网覆盖率达95%以上。

持续推进整乡整村开发扶贫。继续抓好首批11个开发扶贫“整乡推进”试点建设，确保90%以上的扶贫对象有稳定收入来源。各县（市、区）各新选1个乡镇作为第二批“整乡推进”试点，每个试点乡（镇）整合投入资金5000万元以上。启动实施684个贫困村“整村推进”工作，166个贫困村实现脱贫摘帽。深入推进“千企助百村”活动，鼓励和引导非公企业加大对贫困村产业和基础设施建设的帮扶。

（二）推进重大项目建设，增强发展新动能。

稳增长的关键在投资，扩投资的关键在重大项目。必须千方百计保持项目投资较快增长，实施重大项目带动，发挥投资拉动作用。全年完成固定资产投资467亿元。

精心谋划储备项目。紧扣国家、自治区重大战略，推行主管部门专人负责、项目单位主体谋划、聘请专家及时指导的“三位一体”项目谋划工作机制，做好项目策划储备工作。项目库项目滚动保持在350个以上，总投资1100亿元以上，其中，百亿元项目3个以上，十亿元项目10个以上。特别是要抓好项目跟踪落实，我市已编制上报“十三五”重大工程、重大政策、重大项目233项，计划总投资6200亿元。要积极主动向国家和自治区有关部门汇报、衔接，争取得到更多倾斜支持，力争有更多的重大工程、重大政策、重大项目挤进国家和自治区“十三五”规划盘子。

全力以赴推进重大项目建设。提高投资精准性，扩大有效投资，着力推进一批基础设施和重大项目建设。全年自治区和市级层面统筹推进重大项目244项，年度投资149亿元。交通基础设施项目方面，新开工贵阳经河池至南宁客运专线（河池段），贵州瑶山至南丹、天峨至凤山（天峨段）和大化绕城线等二级公路。推进河池至百色、乐业至百色（天峨段）高速公路建设，凤山至凌云、凤山至天峨（凤山段）、三岔至宜州、南丹吾隘至东兰、泗孟至武篆、环江至东江、平果至巴马（坡造）、罗城至柳城（六塘）、都安绕城线、罗城至融水洞头（河池段）等10条二级公路建设；配合推进龙滩水电站升船机等红水河黄金水道航运项目建设。加快黔桂铁路增建二线、柳州经都安至百色、百色经河池至桂林等铁路项目，融水至河池、贺州至巴马、南丹至天峨（下老）等高速公路，环江至金城江一级公路及5条二级公路项目前期工作；抓好巴马通用机场项目前期工作。水利、能源项目方面，开工建设凤山乔音水库扩容工程、110千伏青山（宝坛）送变电工程等项目；做好龙江水资源优化配置工程、河池特色农业高效配水工程、金城江水电站等项目前期工作。产业项目方面，竣工投产南方公司锑银多金属升级改造项目、都安鱼峰西江新型建材、新龙制药公司中成药技改等重大项目，推进南方公司3万吨锡冶炼、大化天寿泉公司母婴天然泉水、宜州桑枝药材综合开发等项目。同时，抓紧推进城镇基础设施、扶贫、教育、卫生等民生项目建设。

抓好项目服务工作。继续深化市四家班子领导联系重大项目工作机制，推行部门联席会、项目推进会以及通报、约谈、督查考评等办法，协调解决项目推进过程中存在的困难和问题。依法依规加快征地工作，为项目建设提供用地保障。积极争取国家、自治区各项投资支持，多渠道筹措建设资金，扩大有效投资。

（三）持续推进新型工业化，加快转型升级步伐。

实施工业强市战略，巩固发展传统产业，培育壮大新兴产业，加快新型工业化跨越发展。

加快园区建设。统筹推进市工业园区、河池•南丹工业园区、河池•东莞工业加工二区（广西宜州经济开发区）、河池•环江工业园区、河池•都安临港工业区等市级及市县联办工业园区建设，每个园区力争引进2个投资5000万元以上工业项目进驻。集中力量推进大任产业园建设，竣工入园二级公路和园区路网一期工程，给排水工程、污水处理厂、110千伏变电站、一期天然气等项目。华远、生富、鑫锋、金兴4家企业实现试产运行。大力支持县级园区建设。全市工业园区（集中区）完成基础设施建设投资3.5亿元以上，工业总产值235亿元以上。

升级改造传统产业。采用新材料、新技术、新工艺，推动有色金属、电力、食品加工、茧丝绸、化工等传统产业改造提升。加快建设生态环保型有色金属产业示范基地，实现新增产值20亿元以上。积极发展清洁能源，加快实施中电国际宜州天然气热电联产项目。推进糖业综合利用和深加工生产，完成环江远丰糖业公司兼并重组、广西博冠纸业蔗渣纸浆板项目建设。扩大丝绸生产规模，延长产业链，提高附加值。推动化工产业扩能增效，实现河化集团技改项目和广维公司生物质化工项目达产达标。五大传统产业实现总产值330亿元，增长13%。

壮大新兴特色产业。建设特色酒业基地，重点推进丹泉、天龙泉、巴马长寿老酒等公司扩能技改项目建设，实现产值35亿元以上。整合提升优质饮用水资源，扩大巴马丽琅、巴马活泉、罗城九千万等知名品牌影响力，做大巴马铂泉、罗城康之源、环江木论思泉等企业，实现产值15亿元以上。大力推进保健养生食品和医药产业开发，实现产值10亿元以上。着力培育碳酸钙骨干企业，建设新型碳酸钙产业基地。

提升服务企业水平。开展企业减负专项行动，推进用电户与发电企业直接交易，建设保税仓，降低企业成本。继续开展“市长企业接待日”活动。深入开展“助保贷、惠企贷”，拓宽企业融资渠道。积极培育后备上市企业，新增上市企业（股权挂牌）5家。发挥公共检测技术服务平台作用，保障企业产品质量。推动企业科技创新，组织3家以上企业申报自治区级企业创新平台。抓好企业培育服务工作，新增规模以上企业15家。倾斜支持强优企业发展，积极打造“百亿南方”“百亿丹泉”，培育一批产值超亿元、十亿元企业。

（四）发展现代农业，促进农业增效农民增收。

重农固本，是安民之基。必须坚持农业重中之重位置，加快转变农业发展方式，构建现代农业产业、生产、经营三大体系，走产出高效、产品安全、资源节约、环境友好的农业现代化道路。全市农林牧渔业总产值246亿元。

抓好现代特色农业（核心）示范区建设。巩固提高都安、南丹、天峨3个自治区级现代特色农业（核心）示范区建设水平，其他县（市、区）建有1个自治区级或市级现代特色农业（核心）示范区，每个乡镇创建1个现代特色农业示范基地。农业核心示范区农民人均纯收入比拓展区高10%以上，比辐射区高15%以上，比所在乡镇高20%以上。大力发展林下经济，每个县（市、区）建立1个林下经济示范基地，全市林下经济面积1025万亩、产值46亿元。

加快发展特色农业。坚持“错位竞争、差别经营、特色发展”理念，扩大“三特”农业种植面积，提高质量和效益。建设“双高”糖料蔗基地2.5万亩，糖料蔗种植面积稳定在90万亩；建立富硒农产品生产基地20个，面积3万亩；食用菌种植1.6亿棒。巩固提升桑蚕、特色水果、油茶等产业发展水平。做大香猪、肉牛、山羊、渔业等特色养殖业，牛羊饲养量、出栏量和内陆大水面开发面积在全区继续领先。继续提升“绿色长寿养生”品牌影响力，新认证“三品一标”产品11个。毫不松懈抓好粮食生产，种植面积稳定在400万亩，总产量101万吨以上。

扶持发展新型农业经营主体。培育农业产业化龙头企业、农民合作社、家庭农场等经营主体，发展多种形式的农业适度规模经营，带动农民致富。抓好农民合作社规范化建设，开展市级示范社认定。发展市级以上农业产业化重点龙头企业达75家，新增农民合作社150家、家庭农场50家。

加强农田水利基础设施建设。新增、恢复和改善灌溉面积19.6万亩，开工或续建水库水源工程11处，抗旱引调提水31处，河流治理26处，建成河堤及护岸工程36公里，完成投资15亿元。提高农业机械装备水平，农作物耕种收综合机械化率43%。

（五）以旅游业为重点，加快发展现代服务业。

立足得天独厚的旅游资源，树立“大旅游”理念，以建设巴马长寿养生国际旅游区为重点，带动百里龙江（“丹金宜罗环”）民族文化生态旅游带协调发展，推进旅游业成为战略性支柱产业，带动现代服务业发展提速、效益提高。

大力发展旅游业。深入实施旅游基础设施建设大会战，打破区域桎梏，完善旅游公共服务体系。着力推动旅游业与其他产业深度融合，打造长寿养生、民族风情、生态休闲、红色之旅等四大旅游品牌，提升“河池长寿地•三姐文化城”旅游整体形象。全年接待国内外游客突破2000万人次，增长15%；旅游总收入突破200亿元，增长17%。

——抓好巴马长寿养生国际旅游区建设。坚持规划引领，完善规划体系，防止盲目开发和低水平重复建设，推进旅游产业转型升级，重点推进巴马命河—水晶宫创5A级景区、赐福小镇、百魔洞，凤山国家地质公园遗迹保护、都安三岛湾国际度假区、东兰红水河第一湾、天峨县城红水河滨水公园等90个项目建设，完成投资10亿元。贯彻实施《广西壮族自治区巴马盘阳河流域生态环境保护条例》，深入开展“两违”综合整治等工作。

——抓好百里龙江民族文化生态旅游带建设。加快金城江珍珠岩、宜州刘三姐故乡风情、罗城武阳江、环江木论世界自然遗产地、南丹洞天酒海等旅游区项目建设。加强与巴马、桂林等国际旅游区对接，促进旅游业发展壮大。

——培育特色旅游品牌。继续开展特色旅游名县创建活动。新增3A级以上景区3个、三星级以上乡村旅游区3个、国家水利风景区1个。加强巴马活泉景区、罗城中天酒庄等工业旅游项目建设。

——完善旅游公共服务设施。重点推进巴马、南丹、天峨、凤山等4个旅游集散中心和巴马、凤山、宜州等3个自驾车营地建设，推进旅游厕所革命，完善旅游标识系统。

加快发展商贸物流业。重点推进汇金国际广场、鸿宇建材城二期工程、城东商业广场和宜州茧丝绸交易中心、东盟国际都安商贸物流城等5个专业市场建设。完善农村市场体系，改造升级5个乡镇农贸市场。继续实施商标品牌战略。积极扩大消费需求，推进教育培训、文化创意和设计服务、运动休闲等领域消费。扩大对外贸易经营，进出口总额达3.84亿美元。

创新发展信息服务业。深入推进“互联网+”和“宽带河池”行动计划，加快推进乡村网络和高速宽带网络建设。市中心城区和各县（市）城区光纤网络覆盖率达98%。发展农村电子商务，鼓励大学毕业生、个体工商户、农民网上创业，推进巴马实施“全国电子商务进农村综合示范县”项目建设。筹建河池百年金城电子商贸物流产业园。各县（市、区）建立农产品电商交易推广平台1个以上。

积极发展金融业。扩大金融资源供给总量，优化供给结构，重点支持实体经济、重大基础设施建设、中小微企业发展。加大普惠金融力度，整合各种贴息政策，创新完善服务方式，统筹推进金融扶贫。支持互联网金融发展。加强金融风险化解处置工作，严厉打击非法集资。继续抓好“引金入河”。年内新增贷款35亿元以上。

稳健发展房地产业。完成固定资产投资22亿元以上。加大住房公积金对住房消费支持力度，不断提高住房公积金支取和贷款的审批效率。加快房地产服务业发展，提高服务管理水平。

（六）加快新型城镇化建设，打造良好宜居环境。

坚持以人为核心的城镇发展理念，强化基础设施建设，依法治理城市，提升人居品位。城镇化率提高1个百分点以上。

统筹城乡规划编制。修编河池市、宜州市总体规划，将东江高铁新区纳入规划范围。完善11个建制镇、18个乡总体规划，启动33个建制镇建成区控制性详细规划。构建金城江宜州城镇化核心区、黔桂走廊城镇带和沿红水河、龙江河城镇带的“一核两带”城镇发展格局。

加快市中心城区建设。抓好城东城西片区建设，竣工芝田和百旺6条道路；开工环卫公寓、人才公寓、市职教中心学校实训楼等项目；推进老虎山、人民厂、铜厂、南桥四大片区棚户区改造、民族公园三期、老年大学、城市社区服务中心、城西水厂取水点搬迁等项目建设。完善旧城改造，竣工任香路、动物园路、星河路、金福路、人行天桥等项目；开工建设翠竹路、辉乐桥（六桥）等项目；加快推进金五小新校区、中心城区地下停车场、市人民医院外科大楼、江北粮食局片区改造等项目建设；推动金城江农贸市场片区改造项目前期工作。完善城区社区基础设施建设，提升社区服务功能，做好小街小巷硬化、亮化、美化改造。

实施大县城战略。每个县（市）打造1个示范新区。完善县城基础设施建设，加快推进县（市）城区管线下地、巷道硬化亮化等市政项目建设。加快县县通天然气工程建设，力争市中心城区、都安、大化用上天然气。实现县城燃气普及率80%以上，污水处理率75%以上，生活垃圾无害化处理率80%以上。

创建特色名镇名村。推进沿江沿河沿路特色城镇建设，打造红水河和龙江河观光休闲、盘阳河健康养生、刘三姐文化旅游、高速交通网沿线民族风情等名城名镇。抓好金城江河池镇，宜州怀远、北山、德胜，罗城龙岸、怀群，环江洛阳、东兴，南丹车河、芒场、六寨，天峨向阳，东兰长乐，都安高岭，大化都阳等15个自治区级百镇示范项目建设；建设宜州福龙、北牙，环江驯乐，南丹中堡、里湖、八圩，天峨八腊，东兰三弄，凤山金牙、平乐、江洲等11个自治区级少数民族乡试点项目；建设环江陈双、南丹同贡、天峨云榜、东兰弄英、巴马达勒、凤山坡心等6个自治区级特色名村项目；建设南丹巴哈、天峨拥里、巴马松屯和达西、都安安乐等5个自治区级乡土特色示范屯试点项目。

提高城市治理水平。提高城市管理执法和服务水平，推动城市管理走向城市治理，促进城市运行高效有序。抓好环境综合整治，持续推进大气污染、环境卫生、交通综合、户外广告、占道经营、饮食摊点排污、文明殡葬、规范养殖等专项整治行动，改善人居环境。坚持“门前三包”常态化管理。

（七）推动绿色发展，建设美丽河池。

以创建国家生态文明先行示范区为目标，实行最严格的环境保护制度，推动生态建设，巩固提升“山青水秀生态美”品牌。

深化生态乡村建设。抓好村屯绿化，建设绿化示范村屯560个。抓好饮水净化，实现全市农村自来水普及率70%以上，初步建成农村饮水安全检测体系。抓好道路硬化，实施29个建制村道路建设，全市建制村道路硬化率100%。

加强生态环境建设。严格划定生态红线和用途保护，加强自然保护区、森林公园、红水河流域、龙江河流域、盘阳河流域等重点区域的生态环境保护。推进“绿满八桂”造林绿化提升、天然林保护、珠江防护林保护等工作，完成植树造林34万亩以上，森林覆盖率68.7%以上。治理岩溶面积380平方公里，治理石漠化面积150平方公里。开展地质灾害防治和地质环境保护工作。

加大环境治理力度。深入开展环保专项整治行动，杜绝重特大环境污染事件发生。加强大气污染防治工作力度，确保市中心城区空气质量持续稳定达到国家二级标准。加快国家级土壤污染防治示范区和龙岩滩库区生态环境保护项目建设。稳步推进重金属污染防治工作。建设一批乡镇生活污水垃圾处理设施项目。

（八）深化改革开放合作，增强发展新活力。

推进结构性改革，以重点领域和关键环节改革突破带动全面改革开放，进一步破除体制机制障碍，释放改革红利，激发发展活力，全面提升经济社会发展竞争力。

推进供给侧结构性改革。大力发展实体经济，增加有效投资，补齐软硬基础设施短板，推动产业转型升级，增加有效供给。降成本方面，落实减税降费让利等政策，降低企业税费、用电、物流等成本。去产能方面，加大力度淘汰落后产能，严格控制增量，防止新的产能过剩。去库存方面，推进户籍制度改革，加快农民工市民化，继续推行“以购代建”模式，稳定房地产市场。

抓好重点领域改革。行政审批制度改革重点是推行政府部门权力清单、责任清单制度，公布投资负面清单。国资国企改革要完善现代企业制度，健全公司法人治理结构，推进外派监事会制度；加大政府投融资体系建设，不断优化政府投融资结构，增强市国投公司、市城投公司等企业融资发展能力，力争融资20亿元以上。财税金融体制改革要推进预算管理改革，规范政府性债务管理，健全预算绩效管理机制；深化农村金融改革，完善面向“三农”和中小微企业的融资担保体系。农村综合改革要全面完成42个试点乡镇农村土地经营权确权登记颁证工作，新增试点乡镇40个以上；积极推进国有林场改革；继续深化供销合作社综合改革试点工作。完成行政（含参公事业）单位公务用车制度改革。统筹推进城市执法体制、不动产统一登记制度、科技、文化、教育、卫生、食品药品监管、收入分配制度等领域改革。

拓展区域开放合作。主动融入“一带一路”发展战略，参与珠江—西江经济带、北部湾经济区开放合作，推动形成通达粤港澳台及沿海地区的人流、物流、资金流、信息流通道。加强桂黔毗邻地区的交流合作，推进天峨•罗甸合作园区项目建设，探索石漠化集中连片特困地区合作发展新途径，推动产业共建合作和生态联保同治。

强力推进招商引资。继续执行招商引资目标责任制。推行招商引资项目代办服务，完善协调推进机制，做好招商项目进驻河池的全方位服务。引进知名企业进行核桃精深加工，延长产业链。强化专题招商，特别是做好工业实体企业招商，谋划旅游项目招商，促进项目落地，履约率90%以上。做好第十三届中国—东盟博览会招商引资。全年招商引资内资到位300亿元，增长11.5%；实际利用外资1.3亿美元，增长8.3%。

发展壮大非公有制经济。完善和落实各项政策措施，实施非公有制强优企业培育计划，奖励非公有制强优企业。升级非公有制企业投诉维权、融资、信息、交流4个服务平台建设，开展“非公经济发展服务月”活动，切实做好服务工作。鼓励和支持社会资本参与国有企业混合所有制改革，参与政府主导的市政、社会事业等基础设施建设。非公有制经济资本投资增长25%以上，新增就业岗位2万个。

（九）全面推进法治建设，巩固社会和谐稳定。

深化平安河池、法治河池建设，运用法治思维和法治方式推进社会综合治理，齐抓共管，群防群治，提升群众安全感和满意度。

创新社会综合治理。深化“天峨经验”“一村一警” 社会治安综合治理模式。深入开展平安创建活动，平安县（市、区）达标率90%以上，平安乡镇（街道）、村（社区）达标率85%以上。完善立体化社会治安防控体系，推进网格化管理工作。全市矛盾纠纷化解率达80%以上。深入推进法治宣传教育，依法严厉打击各类违法犯罪。群众安全感和满意度排名继续位居全区前列。

严格安全监管。落实安全生产“党政同责、一岗双责”和企业主体责任；强化源头治理，严格安全生产行政许可，狠抓重点行业、重点领域安全专项整治和“打非治违”，扎实开展安全生产大检查和安全隐患排查，遏制重特大安全事故发生。稳步推进应急管理体系建设，提高公共突发事件和防灾救灾减灾应急处置能力。加强交通安全管理和消防安全工作。加强食品药品安全监管，严厉打击食品药品生产经营违法行为；深入开展食品安全示范创建活动，争创全区食品安全示范市。加强产品质量安全体系建设，把好产品源头关、准入关、监管关，确保产品质量安全。

促进民族团结进步。认真贯彻落实民族政策，大力开展民族团结宣传教育，深入开展民族团结进步教育基地、示范单位创建工作。继续扶持毛南、仫佬等人口较少民族发展，推动各民族和睦相处、和谐发展。指导巴马办好60周年县庆活动。

积极推动军民融合深度发展。统筹抓好国防建设与地方经济建设。扎实推动国防动员工作。支持部队广泛开展实战化训练演练。推动人防平战结合。不断巩固和发展双拥共建成果，做好拥军优抚安置、民兵预备役等工作，齐心协力争创全国双拥模范城。

（十）切实保障改善民生，共享改革发展成果。

坚持保基本、兜底线、促公平，落实基本公共服务均等化，提高人民群众幸福指数。

加快革命老区振兴发展。大力宣传和弘扬革命老区精神，实施《左右江革命老区振兴规划》，发挥老区资源优势，加快推动老区经济社会发展。加强革命旧址修复、保护和开发，打造红色革命传统教育基地，启动河池拔群干部学院建设，推进爱国主义教育基地和廉政教育基地建设。抓好红色旅游景区景点规划和开发，发挥红色旅游的品牌效益、社会效益和经济效益。

办好教育事业。扩大学前教育资源供给，新建40所、改扩建32所幼儿园，学前三年毛入园率72％。整体提升义务教育均衡发展水平，九年义务教育巩固率89％。推进普通高中多样化、特色化办学，高中阶段教育毛入学率82％。加快推进特殊教育发展。实施现代职业教育发展内涵提升，加快推进市职业教育集团建设，扩大职业教育办学规模。推动数字校园建设，新增70所义务教育阶段学校实现10兆以上宽带接入，50所乡镇中心小学配备多媒体终端设备。落实边远山区教师补贴政策，加强教师队伍建设，完成教师多层次培训2.6万人次。切实关爱留守儿童。妥善解决进城务工人员子女就近入学问题。

推进健康河池建设。创建“中国长寿市”。巩固和完善公共卫生服务均等化，稳步推进10个县（市）28家县级公立医院改革。加强乡镇卫生院能力建设。鼓励社会资本办医。实施中医药壮瑶医药振兴计划。广泛开展爱国卫生运动，市中心城区力争通过自治区卫生城市复审，南丹、凤山力争通过自治区卫生县城验收，环江、天峨、东兰、巴马、大化启动创建工作。抓好妇幼保健、重大传染病防控、艾滋病综合干预等工作，提高人口健康水平。坚持计划生育基本国策，落实一对夫妇可生育两个孩子政策。

促进大众就业创业。推进罗城、环江、天峨、凤山、都安等5个县级就业和社会保障服务中心项目建设，全市139个乡镇（街道）实现公共就业信息网络全覆盖。加大创业创新的资金扶持和政策支持，鼓励高校毕业生、返乡农民工、退役军人、下岗失业人员自主创业。完成职业技能培训、创业培训1万人次，新增城镇就业1.6万人、农村劳动力转移就业5万人次。建设自治区示范性农民工创业园1个、市县级创业孵化基地2个，进驻孵化企业5家以上。

完善社会保障体系。开展“全民参保登记计划”试点工作，城乡养老、城乡医疗保险覆盖率达90%以上，失业、工伤、生育保险覆盖率达到法定群体的80%以上。城乡居民基本养老保险金发放率100%。实现新农合大病保险和居民大病医疗保险全覆盖。整合城乡居民基本医疗保险制度，继续推进医疗保险“一卡通”，实现广西区内异地就医购药即时结算。加强城乡养老服务机构建设，推进市老年公寓综合楼建设，每个县（市、区）建设规范化敬老院2个以上。推进市公墓园区项目和东兰、凤山殡仪服务站建设。建设罗城、南丹、东兰救助管理站和未成年人救助保护中心项目，建成环江、都安未成年人救助保护中心。

繁荣文化体育事业。加强重点文物、非物质文化遗产和特色民俗文化保护开发，加快推进铜鼓文化（河池）生态保护实验区建设。办好第十七届河池铜鼓山歌艺术节。抓好文化市场的日常监管和卫星地面接收专项整治，深入开展“扫黄打非”行动。广泛开展全民健身活动。加强城乡文化体育基础设施建设，加快推进河池民族博物馆、天峨文体中心建设，竣工大化文体中心，启动市新体育场、罗城体育场等文体项目建设。

办好十项惠民实事。一是教育惠民。完成1.3万名幼儿入园补助，落实19万名农村义务教育阶段家庭经济困难寄宿生生活费补助，免除3.9万名高中阶段学生学费，资助2.2万名中职和普通高中贫困生、0.6万名贫困大学新生。二是卫生计生惠民。人均公共卫生服务经费提高到40元／年，儿童一类疫苗报告接种率95%以上，农村孕产妇住院分娩补助率95%以上。扶持农村贫困计划生育家庭小额贷款贴息500户以上，为计划生育家庭购买爱心保险1.5万户以上。三是助残惠民。为残疾人提供康复、托养救助、扶持发展生产、教育资助、重度护理救助等5.4万人次。四是法律服务惠民。推进“一村（社区）一法律顾问”建设，组织律师、基层法律工作者与100个以上村（社区）签订法律顾问协议，办理法律援助案件2200件以上。五是文体惠民。全市群众艺术馆、图书馆、文化馆（站）继续免费开放，8个大型体育场馆免费或低收费开放，新增村级公共服务中心126个、村村通乡镇无线广播台站30个，建成20个数字农家书屋。六是劳动维权惠民。劳动合同签订率90%以上，劳动人事争议案件结案率92%以上，劳动保障监察案件结案率96%以上，实行工资集体协商的企业85%以上。七是落实库区政策惠民。发放大中型水库移民后期扶持资金8700万元、移民口粮补助7600万元，兑现移民子女教育扶持资金1600万元。八是安居惠民。建设保障性住房1.3万套（户），分配入住5000户；完成农村危旧房改造2万户左右。九是农网改造惠民。新建及改造农村电网35千伏线路70.8公里、10千伏线路393.7公里，确保95%以上农户用上电。十是农补惠民。落实农机具购置补贴2500万元；落实农作物良种补贴面积374万亩，发放补贴资金5170万元等强农惠农资金。

三、加强政府自身建设

全面完成今年各项任务，对政府自身建设提出了更高要求。我们要加快建设法治政府、创新政府、廉洁政府和服务型政府，增强政府执行力和公信力，发扬“逢山开路，遇水搭桥”的河池精神，励精图治、砥砺前行，决不辜负全市人民的期望！

（一）提高驾驭全局的能力和水平。强化学习和调查研究，用“五大发展”新理念武装头脑，进一步提高理论水平、政策水平，勇于实践、善于总结，不断提高新常态下政府履职能力。增强工作主动性和预见性，观大势、明大局、循规律，准确把握政策取向，做到心中有数、方法得当。落实重大行政决策专家咨询论证和风险评估制度，发挥政府法律顾问作用，推进行政决策科学化、民主化。

（二）大力推进依法行政。把政府工作纳入法治轨道，严格执行行政裁量权基准制度，公正文明执法，依法开展地方立法工作。自觉接受人大及其常委会的法律监督和工作监督，主动接受政协的民主监督。广泛听取各民主党派、工商联、无党派人士和社会各界的意见建议。保障审计机关依法独立行使审计监督权。加强行政监察，完善纠错问责机制。全面推进政务公开，网络问政实现常态化、规范化，有效利用官方微信、微博、手机客户端等新兴媒介，及时回应社会关切。

（三）切实提高行政效能。持续推进简政放权，放管结合，优化服务，健全联审会审工作机制，简化审批流程，规范审批管理，压缩审批时限，提高办事效率。转变服务理念，规范服务行为，形成为民、利民、便民和亲商、护商、爱商的浓厚氛围，进一步优化经济发展环境。持续强化执行力提升，坚决整治不作为、慢作为、乱作为等突出问题，狠抓工作落实，立说立行，令行禁止。

（四）加强反腐倡廉建设。严格执行中央八项规定、国务院“约法三章”，巩固“三严三实”专题教育成果，持续反“四风”、改作风。落实党风廉政建设 “两个责任”“一岗双责”， 进一步加强政府系统党风廉政建设工作。落实《中国共产党廉洁自律准则》和《中国共产党纪律处分条例》。坚持把纪律和规矩挺在前面，严肃查处发生在群众身边的“四风”和腐败问题。充分利用廉政警示教育基地，深入开展反腐倡廉宣传教育，营造风清气正的发展环境。

各位代表！时代赋予重托，奋斗铸就辉煌。让我们紧密团结在以习近平同志为总书记的党中央周围，在自治区党委、政府和市委的坚强领导下，解放思想，求真务实，敢于担当，主动作为，为实现“十三五”良好开局，建设幸福新河池而努力奋斗！
